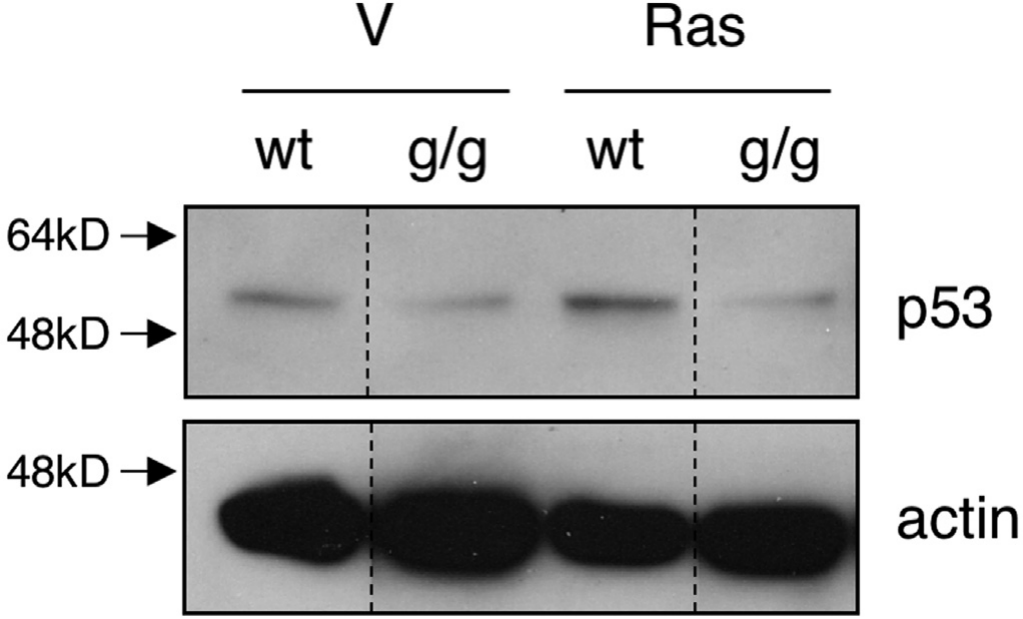# Correction: *Ing1* mediates p53 accumulation and chromatin modification in response to oncogenic stress

**DOI:** 10.1016/j.jbc.2021.100406

**Published:** 2021-03-02

**Authors:** María Abad, Camino Menéndez, Annette Füchtbauer, Manuel Serrano, Ernst-Martin Füchtbauer, Ignacio Palmero

VOLUME 282 (2007) PAGES 31060–31067

During the preparation of the immunoblot image in Figure 3*A*, some lanes with irrelevant samples were deleted. In the composition of the final figure, the panel for the loading control Actin was erroneously assembled, resulting in the duplication of one of the bands. We now include an amended figure with the correct Actin panel, which should replace Figure 3*A*. The legend should be amended to state that: “Dashed lines in A indicate position of lanes removed during figure composition.” This error does not affect any of the conclusions in the article. The authors deeply regret this unintended error and they apologize for any confusion it may have caused to the readers.

**Figure:**